# Consumption of New Zealand Blackcurrant Extract Improves Recovery from Exercise-Induced Muscle Damage in Non-Resistance Trained Men and Women: A Double-Blind Randomised Trial

**DOI:** 10.3390/nu13082875

**Published:** 2021-08-21

**Authors:** Julie E. A. Hunt, Mariana O. C. Coelho, Sean Buxton, Rachel Butcher, Daniel Foran, Daniel Rowland, William Gurton, Heather Macrae, Louise Jones, Kyle S. Gapper, Ralph J. F. Manders, David G. King

**Affiliations:** 1 Department of Nutritional Sciences, Faculty of Health and Medical Sciences, University of Surrey, Guildford GU2 7XH, UK; rachel@naturalfitnessfood.com (R.B.); DanielForan1992@hotmail.com (D.F.); William.gurton@canterbury.ac.uk (W.G.); H.z.macrae2@lboro.ac.uk (H.M.); joneslm95@googlemail.com (L.J.); k.gapper@surrey.ac.uk (K.S.G.); r.manders@surrey.ac.uk (R.J.F.M.); d.g.king@surrey.ac.uk (D.G.K.); 2 Department of Sport and Health Sciences, College of Life and Environmental Sciences, University of Exeter, Exeter EX1 2LU, UK; m.coelho@exeter.ac.uk; 3 SENPRO, Institute of Performance Nutrition, London W1W 7LT, UK; sean@theiopn.com; 4 Environmental Medicine and Science, Institute of Naval Medicine, Gosport PO12 2DL, UK; daniel.rowland106@mod.gov.uk

**Keywords:** exercise-induced muscle damage, recovery, oxidative stress, anthocyanin, New Zealand blackcurrant

## Abstract

Background: Blackcurrant is rich in anthocyanins that may protect against exercise-induced muscle damage (EIMD) and facilitate a faster recovery of muscle function. We examined the effects of New Zealand blackcurrant (NZBC) extract on indices of muscle damage and recovery following a bout of strenuous isokinetic resistance exercise. Methods: Using a double-blind, randomised, placebo controlled, parallel design, twenty-seven healthy participants received either a 3 g·day^−1^ NZBC extract (*n* = 14) or the placebo (PLA) (*n* = 13) for 8 days prior to and 4 days following 60 strenuous concentric and eccentric contractions of the biceps brachii muscle on an isokinetic dynamometer. Muscle soreness (using a visual analogue scale), maximal voluntary contraction (MVC), range of motion (ROM) and blood creatine kinase (CK) were assessed before (0 h) and after (24, 48, 72 and 96 h) exercise. Results: Consumption of NZBC extract resulted in faster recovery of baseline MVC (*p* = 0.04), attenuated muscle soreness at 24 h (NZBC: 21 ± 10 mm vs. PLA: 40 ± 23 mm, *p* = 0.02) and 48 h (NZBC: 22 ± 17 vs. PLA: 44 ± 26 mm, *p* = 0.03) and serum CK concentration at 96 h (NZBC: 635 ± 921 UL vs. PLA: 4021 ± 4319 UL, *p* = 0.04) following EIMD. Conclusions: Consumption of NZBC extract prior to and following a bout of eccentric exercise attenuates muscle damage and improves functional recovery. These findings are of practical importance in recreationally active and potentially athletic populations, who may benefit from accelerated recovery following EIMD.

## 1. Introduction

Strenuous eccentric exercise is known to induce skeletal muscle damage, with prolonged (2–5 days) effects, including reduced muscle strength and range of motion (ROM), increased muscle soreness and swelling, and the leakage of myocellular proteins (e.g., creatine kinase [CK]) into the circulation [1]. Whilst there is evidence to support a potential role for exercise induced muscle damage (EIMD) in the hypertrophic response, a threshold exists, beyond which damage may interfere with the muscle remodeling process [2]. Given that a high degree of EIMD causes pain and impaired muscle function [1,3], excessive damage can reduce an individual’s capacity to train on successive days, dampen the training stimulus and impede adaptation. Furthermore, adverse effects tend to be exacerbated when the exercise is unaccustomed, which could discourage untrained individuals from further participation in exercise activities, preventing repeat exposure and positive training adaptations [4].

The initial phase of EIMD involves structural disruption of the sarcomeres due to mechanical strain, followed by disturbed excitation–contraction coupling and calcium signalling and a proinflammatory response [1]. Leukocyte accumulation contributes to the degradation of damaged muscle tissue through phagocytosis and by releasing proteolytic enzymes and reactive oxygen (ROS) and nitrogen species (RNS) [3]. If left unabated, such derivatives can exceed the antioxidant defense capacity and induce a secondary cascade of muscle fibre disruption, thereby hindering the recovery process [3].

Since inflammation and ROS/RNS derivatives are involved in EIMD, there has been considerable interest in the efficacy of dietary foods/supplements with suggested anti-inflammatory and antioxidant effects to ameliorate EIMD. Plant-derived polyphenolic compounds, such as anthocyanins, show a high endogenous antioxidant capacity [5] and support innate immune defenses [6,7]. Studies that have explored the effects of polyphenolic supplements and rich food/drink sources, such as quercetin [8,9], beetroot [10], blueberry [11], cherry [12,13,14] and pomegranate juice [15,16] have shown equivocal results on recovery from EIMD. The majority find no difference in CK response after exercise [9,10,11,12,13,16], suggesting an inability to protect fibres from secondary muscle damage. This conflicting evidence may be in part attributed to differences in study design. Some have used the contralateral limb to mitigate the repeated bout effect [9,11,12,13,14] but carryover to the non-exercising limb may confound results [17,18], and therefore a between-subject research design is preferable. Variation in mode, intensity and duration of exercise to elicit muscle damage is also apparent. EIMD is greater and/or recovery is slower following single joint upper body exercise [19,20], when contractions are performed at high intensity [21] and at a long muscle length [22]. Finally, the type, dose and duration of polyphenolic supplementation is likely to influence the efficacy of the recovery intervention. Supplementing before exercise may effectively prime the cell (up-regulating antioxidant capacity) to cope with the initial increase in ROS, while continued supplementation in the recovery period may attenuate ROS induced secondary damage [23]. Blackcurrants possess a high inherent antioxidant capacity compared to most other fruits, which is attributed to their high anthocyanin and vitamin C content [24,25]. Consumption of anthocyanin-rich New Zealand blackcurrants (NZBC) extract exerts positive physiological effects on the cardiovascular [26,27,28], metabolic [29] and immune systems [30,31], but few studies have examined their capacity to attenuate traditional symptoms of EIMD (e.g., strength, soreness, ROM, CK concentration) [23]. Given the close relationship between plasma anthocyanin bioavailability and functional efficacy, it may be beneficial to perform exercise 1–2 h after the consumption of supplements/foods, to coincide with peak plasma anthocyanin levels [32,33,34], in an attempt to maximize adaptive cellular events and facilitate recovery. Indeed, recent studies have found acute consumption of NZBC extract 1 h prior to exercise facilitates recovery from exercise-induced oxidative stress and preserves circulating neutrophil function [35], and when consumed daily, promotes a protective antioxidant/anti-inflammatory cellular environment that supports positive adaptative processes [36].

The purpose of this study was to investigate the effects of NZBC extract on indices of muscle damage and recovery following a bout of strenuous isokinetic resistance exercise. We hypothesized that given its high anthocyanin content, NZBC would protect against exercise-induced inflammation and oxidative stress, reducing the extent of secondary muscle damage and facilitate a faster recovery of muscle function.

## 2. Materials and Methods

### 2.1. Participants

Twenty-seven healthy and non-resistance trained males and females volunteered to participate in the study. Participants were recruited from student and staff cohorts at the University of Surrey by word of mouth, email and social media displays. Participants were included if they were aged between 18–45 years, had a healthy BMI (19–29.9 kg/m^2^) and did not meet physical activity guidelines for resistance exercise frequency (<2 sessions per week). Participants were excluded if they (i) smoked, (ii) took medication (excluding contraception), (iii) had a BMI ≥ 30 kg/m^2^, (iv) had hypertension, (v) had a history of musculoskeletal upper limb injuries, (vi) performed resistance exercise more than twice per week and/or (vii) used dietary supplements that could influence muscle recovery or function (i.e., protein supplements, antioxidants). All participants completed a pre-participation health questionnaire, and were informed of the purpose, risks and discomforts associated with the investigation before giving written and informed consent. The study was conducted in accordance with the Declaration of Helsinki, and the protocol was approved by Surrey University Research Ethics Committee (UEC/2015/112/FHMS) prior to the first participant enrolment (10 June 2016) and was retrospectively registered on the ClinicalTrials.gov (NCT05010057) database (17 August 2021). Participant characteristics and descriptive statistics are listed in Table 1.

### 2.2. Study Design

The study used a double-blind, randomised, placebo-controlled, parallel design to investigate the effects of NZBC extract on indices of muscle damage and recovery following a bout of strenuous isokinetic resistance exercise. Using a sealed envelope randomised block procedure, familiarised participants received either the NZBC extract (*n* = 14) or the placebo (PLA) (*n* = 13) for 12 days. On day 8, participants performed maximal concentric and eccentric contractions of the biceps brachii muscle group on an isokinetic dynamometer (CSMi Humac Norm, Massachusetts). Muscle soreness (using a visual analog scale), muscle function (via measures of maximal voluntary contraction [MVC]), ROM and serum CK were assessed before (0 h) and after (24, 48, 72 and 96 h) exercise.

Criterion measures were performed on days 8–12 at a similar time in the morning (between 7–10 am ± 1 h) after a 10 h overnight fast. Participants were asked to avoid taking non-steroidal anti-inflammatory drugs (NSAIDs) for the duration of the study period and abstain from exercise and alcohol consumption between days 7 and 12. A 6-day food diary was started 24 h prior to the muscle damage exercise bout and continued for 96 h (day 7–12). This was analysed using Nutritics software to determine macronutrient distribution and total energy intake during the study period. In addition, daily anthocyanin intake was estimated using the Phenol-Explorer database [37]. A physical activity diary was used to check compliance with instructions to avoid exercise.

### 2.3. Preliminary Measures and Familarisation Session

Participants attended a preliminary visit 2–3 weeks prior to experimental trials to determine anthropometric measures, establish settings on the isokinetic dynamometer (Cybex NORM^®^, Humac, CA, USA) and practice the protocols for assessing strength (MVC) and inducing muscle damage. Stature and body mass were measured using a portable stadiometer (Seca 213, Seca GmbH, Hamburg, Germany) and digital column scales (Seca 799, Seca GmbH, Hamburg, Germany), respectively. The isokinetic dynamometer was set up according to manufacture guidelines for elbow flexion, with the dynamometer orientated at 0° tilt, 5° rotation and position 4 for height. The position of the seat was adjusted for each individual to align the lateral epicondyle with the dynamometer axis of rotation. This set-up was recorded and replicated on subsequent visits. Participants practiced MVCs in accordance with the protocol below and completed ten repetitions of the muscle damage protocol to confirm they were comfortable with the range of motion (5° to 135° of elbow flexion). The latter was performed at submaximal intensity (RPE 5, Borg CR10 scale) to avoid repeated bout effect.

### 2.4. Supplement Dosing (Day 1–12)

Familiarised participants received twelve NZBC extract (300 mg active cassis containing 105 mg of anthocyanins, i.e., 35–50% delphinidin-3-rutinoside, 5–20% delphinidin-3-glucoside, 30–45% cyanidin-3-rutinoside, 3–10% cyanidin-3-glucoside) (Health Currancy Ltd. [Camberley, UK]/CurraNZ Ltd. [Auckland, New Zealand]) or placebo (300 mg microcrystalline cellulose M102) capsules, and were instructed to consume one capsule every morning. On day 8, participants were instructed to consume their supplement 1 h before performing the muscle damage exercise protocol.

### 2.5. Muscle Damage Exercise Protocol (Day 8)

Following a warm-up and measures of MVC on the isokinetic dynamometer (see description below) the participants performed 4 × 15 repetitions of maximal concentric and eccentric contractions of the biceps brachii muscle group in the dominant arm. Participants were given a 1-min recovery period between sets. The angular velocity was set at 45°/s for concentric actions and 60°/s for eccentric actions. The ROM was standardized from 5° to 135° of elbow flexion. Participants were instructed to perform the repetitions ‘as hard as they can’ and investigators gave verbal encouragement throughout. Additional MVC assessments were performed immediately post-exercise (0 h) to provide insight into the fatiguability of the exercise protocol. 

### 2.6. Criterion Measures (Days 8–12)

Criterion measures were performed before muscle damage exercise on day 8, and at the same time on days 9, 10, 11 and 12 within a ± 1 h window, reflecting 24, 48, 72 and 96 h post-exercise timepoints.

Elbow flexor muscle soreness was measured using a visual analogue scale (VAS). Participants were asked to extend their dominant arm and rate their perceived pain on the horizontal scale, from ‘no soreness’ (0 mm) on the left anchor point to ‘extremely sore’ (100 mm) on the right. Pain was identified by measuring the distance from the left anchor point (0 mm).

Elbow range of motion (ROM) was determined using a two-arm goniometer. The goniometer axis was placed over the lateral epicondyle, and the stationary and moving arm were aligned with the humerus (centre of acromion process) and radius (centre of styloid process), respectively. Participants were asked to maximally flex the elbow joint and then relax their arm until the point of discomfort, whilst ensuring their upper arm stayed vertical and in contact with the side of their body. The joint angle at maximal flexion and extension was measured 3 times, and total ROM was calculated before being averaged.

Participants’ mid-upper arm circumference (MAC) was measured at the midpoint of the distance from the acromion process (acromiale) to the olecranon process (radiale). Three measures were made and the average recorded.

For serum creatine kinase, 4 mL blood samples were obtained from the antecubital vein prior to eccentric exercise (day 8) and at 24, 48, 72 and 96 h post-exercise (day 9, 10, 11 and 12). Samples were centrifuged for 10 min at 1000× *g* at 4 °C. Analysis of serum CK concentrations was conducted by an external pathology lab (Berkshire and Surrey Pathology Services) and performed with CK assay kits using the ADVIA^®^ 1800 Clinical Chemistry System (Siemens Healthcare Ltd, Camberley, UK).

For muscle function, participants performed a warm-up involving low load resistance exercise (1 × 20 reps of single arm bicep curl at ~5 RPE (Borg CR10 scale) and a stretching routine of the major muscle groups (latissimus dorsi, pectorals, rhomboids, deltoids, biceps, triceps, wrist flexor and extensor) of the upper extremity. They then lay on the isokinetic dynamometer with their dominant arm secured at their side in 90° elbow flexion. Participants performed 3 submaximal isometric contractions as a warm-up, followed by 3 maximal efforts held for 5 s interspaced with 60 s recovery. Isometric MVC was determined from the highest peak torque (N-m) from the best trial (out of 3).

### 2.7. Statistical Analysis

Statistical analysis was performed using the Statistical Package for the Social Sciences (SPSS 24, IBM, Armonk, NY, USA). Shapiro–Wilks tests were utilized to test the data for normality, and when this assumption was violated, log-transformations were attempted. Levene’s tests were used to verify homogeneity of variance and Mauchly’s tests to assess sphericity. When the sphericity assumption was violated, Greenhouse–Geisser corrections were performed. Independent sample t-tests were performed to determine if there were significant differences between groups at baseline. Repeated measures ANOVAs were performed for mid-arm circumference, ROM and MVC. Friedman and Mann–Whitney U tests were used to analyze perceived soreness and CK results. For soreness, post-hoc analysis with Wilcoxon signed-rank tests was conducted with a Bonferroni correction to control for type I errors. All data are expressed as mean ± standard deviation, unless stated otherwise.

## 3. Results

### 3.1. Participants

The characteristics of participants in the NZBC and PLA group are displayed in Table 1. There were no differences in baseline variables between groups. All participants completed daily supplement logs and reported consumption of their capsules in the morning as instructed, suggesting 100% compliance. Analysis of the food diaries confirmed there was no difference in total energy intake, consumption of macronutrients and anthocyanin between groups (Table 2).

### 3.2. Muscle Function

**Figure 1:**
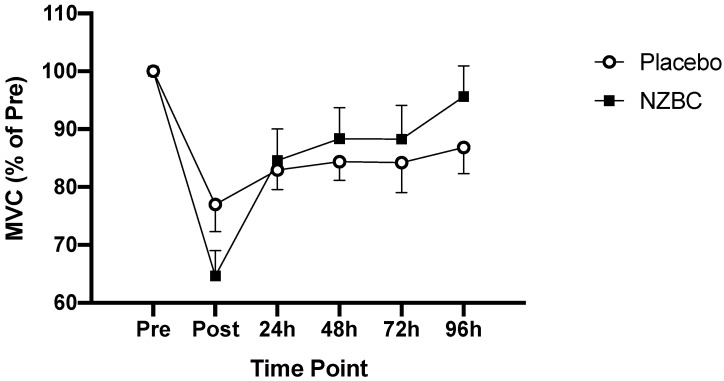
Change in maximal voluntary contraction of the elbow flexor muscles following eccentric exercise (percentage of pre-exercise strength) in the PLA and NZBC groups. Values are means ± SEM.

Figure 1 displays the change in MVC following the muscle damage protocol (2-way ANOVA main effect for time, *p* < 0.001). Post-hoc Bonferroni analysis confirmed MVC was significantly lower immediately, 24 h, 48 h and 72 h post-exercise (*p* < 0.05) but participants had regained their baseline strength by 96 h post-exercise (*p* = 0.251). There was no group (2-way ANOVA main effect for group, *p* = 0.399) or interaction effect (2-way ANOVA group * time, *p* = 0.162) for MVC. However, in the NZBC group, only transient reductions in MVC were observed immediately following the eccentric exercise (mean difference; 15.5 ± 9.8 Nm, *p* = 0.001) with no difference in strength measures at 24 h (6.7 ± 8.3 Nm, *p* = 0.245) 48 h (5.9 ± 9.3 Nm, *p* = 0.538), 72 h (5.6 ± 8.0 Nm, *p* = 0.554) and 96 h (3.5 ± 10.1 Nm, *p* = 0.898) post-exercise vs. baseline (1-way ANOVA with post-hoc Bonferroni analysis, *p* < 0.05). When post-exercise MVC was expressed relative to baseline (%), an interaction effect was observed (2-way ANOVA group * time, *p* = 0.035).

### 3.3. Creatine Kinase

**Figure 2:**
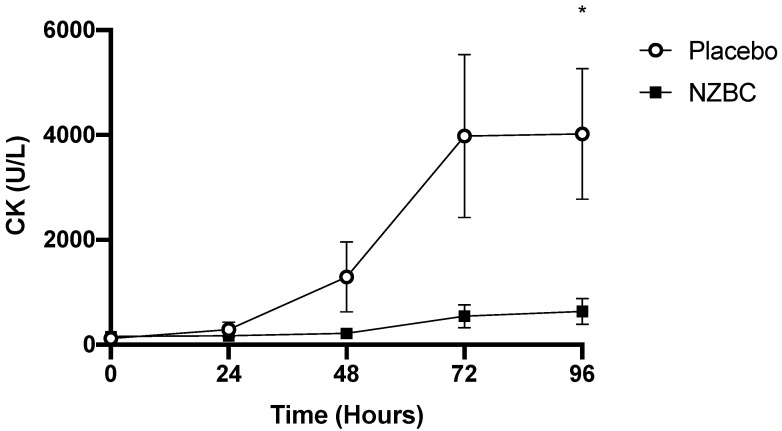
Creatine Kinase (CK) before and after eccentric exercise in the PLA and NZBC groups. Values are means ± SEM, * significant difference between groups (*p* < 0.05).

Figure 2 displays the changes in serum CK concentration over the course of the experimental period. CK significantly increased after the muscle damage protocol in the PLA group (Friedman Test, *p* = 0.008) but remained unchanged in the NZBC group (Friedman Test, *p* = 0.798). Post-hoc Wilcoxon signed rank analysis with Bonferroni correction confirmed CK was significantly elevated above baseline in the PLA group at 96 h post-exercise (*p* = 0.003). Furthermore, serum CK levels in the NZBC group displayed a trend toward being lower at 72 h (Mann-Whitney U test, NZBC: 542 ± 818 UL vs. PLA: 3977 ± 5392 UL, *p* = 0.080) and were significantly lower at 96 h post-exercise (Mann–Whitney U test, NZBC: 635 ± 921 UL vs. PLA: 4021 ± 4319 UL, *p* = 0.040).

### 3.4. Muscle Soreness

**Figure 3:**
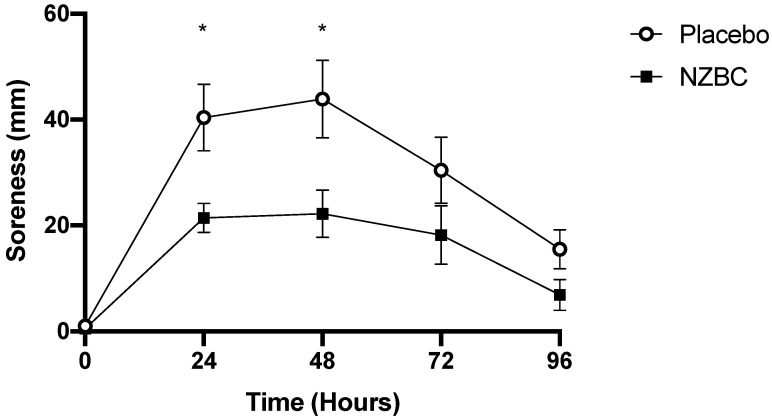
Muscle soreness before and after eccentric exercise in the PLA and NZBC groups. Values are means ± SEM, * significant difference between groups (*p* < 0.05).

Participants in both groups reported a significant change in muscle soreness over time (Friedman Tests, *p* < 0.001, Figure 3). An increase in muscle soreness was observed 24 h after the muscle damage protocol, peaking at 48 h and decreasing thereafter (Post-hoc Wilcoxon signed rank analysis with Bonferroni correction, *p* < 0.01). A Mann–Whitney U test confirmed participants in the NZBC group experienced reduced muscle soreness compared to the PLA group at 24 h (NZBC: 21 ± 10 mm vs. PLA: 40 ± 23 mm, *p* = 0.018) and 48 h (NZBC: 22 ± 17 vs. PLA: 44 ± 26 mm, *p* = 0.025) post-exercise.

### 3.5. Range of Motion

Participants in both groups experienced a significant change in their elbow ROM over time (Friedman Tests, *p* < 0.01). A decrease in elbow ROM was evident from 24 h post-exercise (Post-hoc Wilcoxon signed rank analysis with Bonferroni correction, *p* < 0.001). At 96 h post-exercise, ROM had recovered in the NZBC group but remained suppressed in the PLA group compared to baseline (Table 3). However, there was no difference between groups at any time point (Mann–Whitney U tests, *p* > 0.05, Table 3).

### 3.6. Mid-Arm Circumference

MAC significantly changed in response to the muscle damage protocol (2-way ANOVA main effect for time, *p* = 0.026, Table 3). Post-hoc analysis with Bonferroni correction showed MAC increased from baseline at 48 h post-exercise (post-hoc t-test with Bonferroni correction, *p* = 0.01). There was no group (2-way ANOVA main effect for group, *p* = 0.856) or interaction effect (2-way ANOVA group * time, *p* = 0.116).

## 4. Discussion

The purpose of this study was to investigate the effects of NZBC extract on indices of muscle damage and recovery following a bout of strenuous isokinetic resistance exercise. This is the first study to observe that 12-day intake of NZBC extract by non-resistance trained individuals results in faster recovery of muscle function (maximal isometric torque), attenuated muscle soreness (at 24–48 h) and serum CK concentration (at 96 h) following EIMD. Our results suggest consumption of NZBC extract prior to and following a bout of eccentric exercise attenuates muscle damage and improves functional recovery. These findings are of practical importance in recreationally active and potentially athletic populations, who may benefit from accelerated recovery following EIMD.

Muscle fibre disruption occurs following strenuous exercise, especially if the participant is unaccustomed to the activity, and it involves eccentric muscle contractions. It is commonly characterized by a decrease in muscular strength and ROM, localised inflammation and muscle soreness, and elevated levels of intramuscular proteins (e.g., CK). in the blood. We observed such changes in criterion measures in both participant groups, which suggests our isokinetic resistance exercise protocol evoked a degree of muscle fibre disruption. However, in support of our hypothesis, we found that consumption of NZBC extract was able to protect the skeletal muscle and reduced the extent of EIMD.

A reduction in maximal isometric torque is regarded as the most valid indicator of EIMD [38]. Muscle strength decreased by ~29% immediately after exercise and was restored within 96 h, in agreement with previous studies implementing a similar eccentric elbow flexor exercise protocol [15]. Although the magnitude of the decrease in MVC was the same between groups, the rate of MVC recovery was accelerated in the NZBC group. This may reflect a reduced magnitude of muscle damage as described by Chen et al. [39]. The initial damage and strength loss following lengthening muscle actions is thought to occur because of both mechanical strain and oxidative stress [40]. Consumption of blackcurrant anthocyanins may alleviate oxidative stress and facilitate recovery [30]. Indeed, a recent study has shown consumption of blackcurrant anthocyanin-rich extract (BAE) 1 h prior to exercise can reduce plasma protein carbonyl levels and plasma oxidative capacity at 2 and 6 h post-exercise compared to the placebo group [35]. Authors speculated that the increase in plasma anthocyanins as a result of BAE consumption, activated cellular redox-sensitive signaling pathways (e.g., Nrf2/ARE transcription), causing the up-regulation of cellular antioxidant systems and subsequently lowering oxidative stress indices during exercise recovery. Although in the present study we did not perform mechanistic experiments it seems plausible that the consumption of NZBC extract increased cellular antioxidant defense capacity, thus reducing initial muscle damage and promoting tissue repair.

Primary muscle damage initiates a cellular repair signaling pathway. Neutrophils and monocytes migrate to the damaged tissue, and their accumulation promotes the degradation of cellular debris through phagocytosis and the release of ROS and RNS [1]. The problem arises when the degradation is not exclusive to cellular debris, but also affects previously uninjured muscle. This is considered the second phase of EIMD and leads to delayed onset of muscle soreness (DOMS) and increased CK release. DOMS peaked in our participants at 48 h, with pain persisting until 72 h, which is consistent with previous observations [1]. Participants who consumed NZBC extract reported less pain with active elbow extension than subjects who consumed the placebo. Muscle tenderness is thought to occur as a result of oedema. Specifically, disruptions to the extracellular matrix (ECM) cause an increase in interstitial inflammatory mediators, which interact with nociceptors in the muscle to trigger the pain response [41]. The consumption of BAE has been previously shown to have anti-inflammatory effects [30], although the underlying mechanism is unknown. Improvements in blood flow as a result of BAE consumption may support rapid neutrophil influx and outflux. Furthermore, BAE consumption is found to enhance neutrophil antioxidant capacity and preserve functionality against exercise-induced oxidative stress [35]. By collectively improving blood flow and preserving neutrophil function (minimizing dysregulation (e.g., delated apoptosis)), it may reduce the accumulation of neutrophils, the release of their cytotoxic products and further local tissue damage. NZBC consumption may therefore have attenuated further disruption to the ECM, reduced inflammation (and oedema) and the nociceptor response. Whilst we observed an increase in MAC following exercise indicating swelling, there was no difference between groups. It is likely that circumference measurements are not sensitive enough to assess oedema particularly when related to micro-rather than macro-trauma. Furthermore, localized swelling may have been more prominent at other points/lengths of the muscle and not captured by this mid-arm measure. In line with similar levels of oedema, we observed no difference in relaxed elbow ROM between groups, indicating a comparable level of muscle and/or joint stiffness, as observed previously in tart cherry studies [13,14,16].

We observed a significant elevation in serum CK post-exercise in the placebo group. This response is similar to that reported in previous studies utilising a similar exercise protocol (eccentric elbow flexion) in non-resistant trained population [9,42]. We noted a large inter-individual variation in the CK response to the muscle damage protocol in the placebo group. Whilst this is consistent with the literature [43], it is interesting that the variability between individuals is significantly smaller in the NZBC group. We speculate that this is due to the free radical scavenging activity and reducing power of black currant extract [44]. Indeed, CK levels were significantly reduced in the NZBC group at 96 h post-exercise suggesting attenuation of secondary muscle damage caused by inflammatory amplifiers and ROS/RNS. This is in contrast to other studies investigating different antioxidant-rich foods/supplements that found no reduction in circulating CK levels relative to controls/placebos [11,12,45]. By supplementing with NZBC before (8 days) and in the days following (4 days) strenuous exercise, we may have increased anthocyanins bioavailability and effectively primed the cell (up-regulating antioxidant capacity) to neutralize the initial (exercise-induced) and continued release of ROS (in damaged myofibres), and attenuated further disruption of the sarcolemma by oxidative reactions and additional leakage of CK [23]. Similarly, consumption of black currant nectar (BCN) reduced CK levels at 48 and 96 h post eccentric knee extension exercise (3 × 10 sets @ 115% of IRM) [23]. This was preceded by a reduced elevation in IL-6 at 24 h post-exercise in the BCN vs. placebo group, indicating a dampened inflammatory response.

The inflammatory response to myodamage can have either a beneficial (i.e., contribute to growth processes) or detrimental (i.e., exacerbate the initial mechanical damage) effect on muscle function depending on the magnitude of the response [2,46]. Excessive or inappropriate timing of supplements (e.g., antioxidant, anti-inflammatory) that significantly dampen oxidative stress during exercise may prevent adaptive events and be detrimental to the recovery process [47]. The impact of prolonged daily NZBC extract consumption on the hypertrophic response to resistance training is not known. Early indications appear positive; daily consumption of BAE for 5 weeks promotes antioxidant/anti-inflammatory cellular events that facilitate exercise recovery [36] but the influence on training adaptations are yet to be examined.

This is the first study to investigate whether NZBC extract consumption can attenuate traditional symptoms of resistance based EIMD. The strengths of this study include the use of an isokinetic dynamometer to better control the stimulus for muscle damage and the adoption of a double-blind placebo-controlled, parallel design, which is preferable to avoid the repeated bout effect. However, this study also has several limitations that must be considered when interpreting the findings. We only assessed indirect markers of muscle damage and it would have been valuable to determine if histological and/or biochemical differences were observed. We did not measure systemic markers of inflammation (IL-6, TNF-a, IL-8) as these do not adequately reflect the inflammatory response to eccentric exercise. Indeed, exercise-induced changes in TNF-α and IL-8 concentrations are much greater in the muscle and interstitial cells than the circulation [48,49]. We observed some high CK responders, based on a lower limit for exertional rhabdomyolysis (CK ≥ 1000 U/L [50]) and yet our changes in muscle function were comparatively moderate. A dissociation between strength loss and histological evidence of fibre damage (i.e., greater histopathology relative to the magnitude of strength loss) has been observed previously [41,51,52]. Alternatively, MVC measures may not have been sensitive enough to detect performance changes. Torque is the product of force and movement arm, the latter being joint angle dependent. MVC measurements were performed at the same joint angle (90°), which ensured valid comparisons within and between individuals, but it may have been better to assess muscle force output at a longer muscle length or perform measures of dynamic strength, to better simulate the movement pattern associated with the exercise task. Future studies should include measures of dynamic and isometric performance. Regretfully, we did not monitor which phase of the menstrual cycle our female participants were in at the time of testing. Whilst hormone fluctuations throughout the menstrual cycle may affect EIMD in terms of strength loss and DOMS, they are unlikely to explain the difference we have observed in CK [53]. Finally, whilst our supplementation was appropriately timed (ingested within the critical window of 1–2 h pre-exercise), we acknowledge that anthocyanin intake should be tailored to individual needs. We provided an absolute rather than relative (mg/kg) dose of anthocyanins. The daily consumption of 3 g NZBC extract contained 105 mg anthocyanins, which retrospectively equated to 1.6 ± 0.3 mg/kg. This is believed to be the minimal effect dose [35] and has been shown to facilitate recovery from exercise-induced oxidative stress [30,35]. Future studies should manipulate the administered NZBC amount based on participants body weight and total anthocyanins (mg) in the NZBC extract to ensure all participants obtain more than the minimal effect dose.

## 5. Conclusions

In conclusion, this study demonstrates that consuming an anthocyanin-rich black currant extract 8 days prior to and for 4 days following strenuous eccentric exercise resulted in faster recovery of muscle function and attenuated muscle soreness and serum CK activity. Prolonged supplementation with NZBC has been shown by others to facilitate exercise recovery [36] but the capacity to promote (or mitigate) training-induced adaptations is unknown. Future research should examine the effect of chronic daily NZBC supplementation on training capacity (performance on successive days) and the hypertrophic response to resistance exercise in recreationally active and athletic populations. Muscle biopsies should be acquired to investigate exercise-induced inflammation and oxidative stress to examine the mechanisms and confirm the extent of reduced secondary muscle damage and facilitated recovery associated with daily consumption of NZBC extract.

## Figures and Tables

**Table 1:** Participant characteristics and descriptive statistics between the NZBC (*n* = 14) and PLA groups (*n* = 13).

	NZBC	Placebo	*p* Value
Gender	10 women, 4 men	9 women, 4 men	
Age (yrs)	24 ± 2	23 ± 2	0.272
Stature (cm)	170 ± 9	164 ± 11	0.175
Body Mass (kg)	67.9 ± 15.7	59.0 ± 7.9	0.120 *
BMI (kg/m^2^)	23.4 ± 3.5	21.8 ± 2.3	0.204
Baseline MVC (Nm)	42.2 ± 18.3	35.8 ± 13.9	0.299
Baseline CK (UL)	118 ± 80	155 ± 99	0.487

Values are means ± SD. *p* value denotes Independent T-test or * Mann-Whitney U outcome. NZBC, New Zealand Blackcurrant; PLA, Placebo; MVC, maximal voluntary contraction; CK, creatine kinase.

**Table 2:** Dietary analysis for NZBC (*n* = 14) and PLA groups (*n* = 13).

	NZBC	Placebo	*p* Value
Energy (kcal day^−1^)	1880 ± 905	1617 ± 408	1.000 *
Carbohydrate (g day^−1^)	221 ± 110	182 ± 53	0.305
Protein (g day^−1^)	78 ± 22	69 ± 22	0.484
Fat (g day^−1^)	68 ± 25	74 ± 46	0.784
Anthocyanin (mg day^−1^)	24 ± 28	38 ± 33	0.279 *

Values are means ± SD. *p* value denotes Independent *t*-test or * Mann–Whitney U outcome.

**Table 3:** Elbow range of motion (ROM) and mid arm circumference (MAC) in NZBC and Placebo groups before and after muscle damage protocol.

Variable	Group	0 h	24 h	48 h	72 h	96 h
ROM (°)	NZBC	150 ± 21	139 ± 34 *	139 ± 31 *	140 ± 25 *	142 ± 26
	PLA	147 ± 20	134 ± 26 *	134 ± 26 *	136 ± 22 *	136 ± 20 *
MAC (cm)	NZBC	28.6 ± 4.2	29.0 ± 4.2	29.1 ± 4.2	28.6 ± 4.3	28.6 ± 4.1
	PLA	27.9 ± 2.9	28.0 ± 2.9	28.3 ± 2.7	28.6 ± 2.6	28.6 ± 2.4

ROM, range of motion; MAC, mid-arm circumference; NZBC, New Zealand Blackcurrant; PLA, Placebo. Post-hoc Wilcoxon signed rank analysis with Bonferroni correction * significantly different to pre-exercise (0 h) (*p* < 0.05).

## Data Availability

The data presented in this study are available on request from the corresponding author. The data are not publicly available due to ethical restrictions.
